# Isolated REM Sleep Behaviour Disorder—Is Screening Possible?

**DOI:** 10.1111/jsr.70109

**Published:** 2025-06-03

**Authors:** Matteo Cesari, Andreas Brink‐Kjaer, Emmanuel During, Wolfgang Ganglberger, Oriella Gnarra, Bei Huang, Michael Sommerauer, Pietro Luca Ratti, Irene Rechichi, Yun Kwok Wing, Ambra Stefani

**Affiliations:** ^1^ Department of Neurology Medical University of Innsbruck Innsbruck Austria; ^2^ Department of Health Technology Technical University of Denmark Kgs. Lyngby Denmark; ^3^ Department of Neurology Icahn School of Medicine at Mount Sinai New York New York USA; ^4^ Division of Pulmonary, Critical Care and Sleep Medicine Icahn School of Medicine at Mount Sinai New York New York USA; ^5^ Department of Neurology Beth Israel Deaconess Medical Center Boston Massachusetts USA; ^6^ Harvard Medical School Boston Massachusetts USA; ^7^ Department of Health Science and Technologies Institute of Robotics and Intelligent Systems, ETH Zurich Zurich Switzerland; ^8^ Li Chiu Kong Family Sleep Assessment Unit, Department of Psychiatry Faculty of Medicine, the Chinese University of Hong Kong Hong Kong China; ^9^ Li Ka Shing Institute of Health Sciences, Faculty of Medicine The Chinese University of Hong Kong Hong Kong China; ^10^ Center of Neurology, Department of Parkinson, Sleep and Movement Disorders University of Bonn Bonn Germany; ^11^ German Centre for Neurodegenerative Diseases (DZNE) Bonn Germany; ^12^ Nuffield Department of Clinical Neurosciences University of Oxford Oxford UK; ^13^ Clinical Neurophysiology Unit Clinical Neurosciences Division, Oxford University Hospital NHS Foundation Trust Oxford UK; ^14^ Department of Control and Computer Engineering Politecnico di Torino Turin Italy

**Keywords:** iRBD, prodromal alpha‐synucleinopathy, RBD, screening tool

## Abstract

Rapid eye movement (REM) sleep behaviour disorder (RBD) is a parasomnia characterised by the loss of muscle atonia during REM sleep and dream‐enacting behaviours. In its isolated form (iRBD) it is widely recognised as an early stage of alpha‐synucleinopathies. Early identification of patients with iRBD allows for timely interventions, risk mitigation and potential inclusion in clinical trials aimed at disease modification. Effective screening tools, including questionnaires, automatic analyses of video‐polysomnography, actigraphy, nearables and biological markers, can facilitate diagnosis and monitoring. Incorporating routine screening into clinical practice may enhance early detection and improve long‐term patient outcomes. This manuscript presents the latest developments in screening tools for the identification of patients with iRBD and discusses their advantages and drawbacks, highlighting paths for future research and applications.

## 1 Introduction

Rapid eye movement (REM) sleep behaviour disorder (RBD) is a parasomnia characterised by dream enactment and the lack of muscle atonia during REM sleep (American Academy of Sleep Medicine 2023). When RBD occurs in the absence of overt neurological diseases which are known to be associated with it, it is classified as isolated RBD (iRBD). This condition is widely recognised as an early stage of alpha‐synuclein‐related neurodegenerative diseases, which include Parkinson's disease (PD), dementia with Lewy bodies (DLB) and multiple system atrophy (MSA) (Galbiati et al. 2019; Högl et al. 2022; Postuma et al. 2019; Stefani, Trenkwalder, et al. 2023). Consequently, patients with iRBD represent an optimal target population for initiating neuroprotective and disease‐modifying clinical trials (Postuma 2022; Videnovic et al. 2020).

**FIGURE 1:**
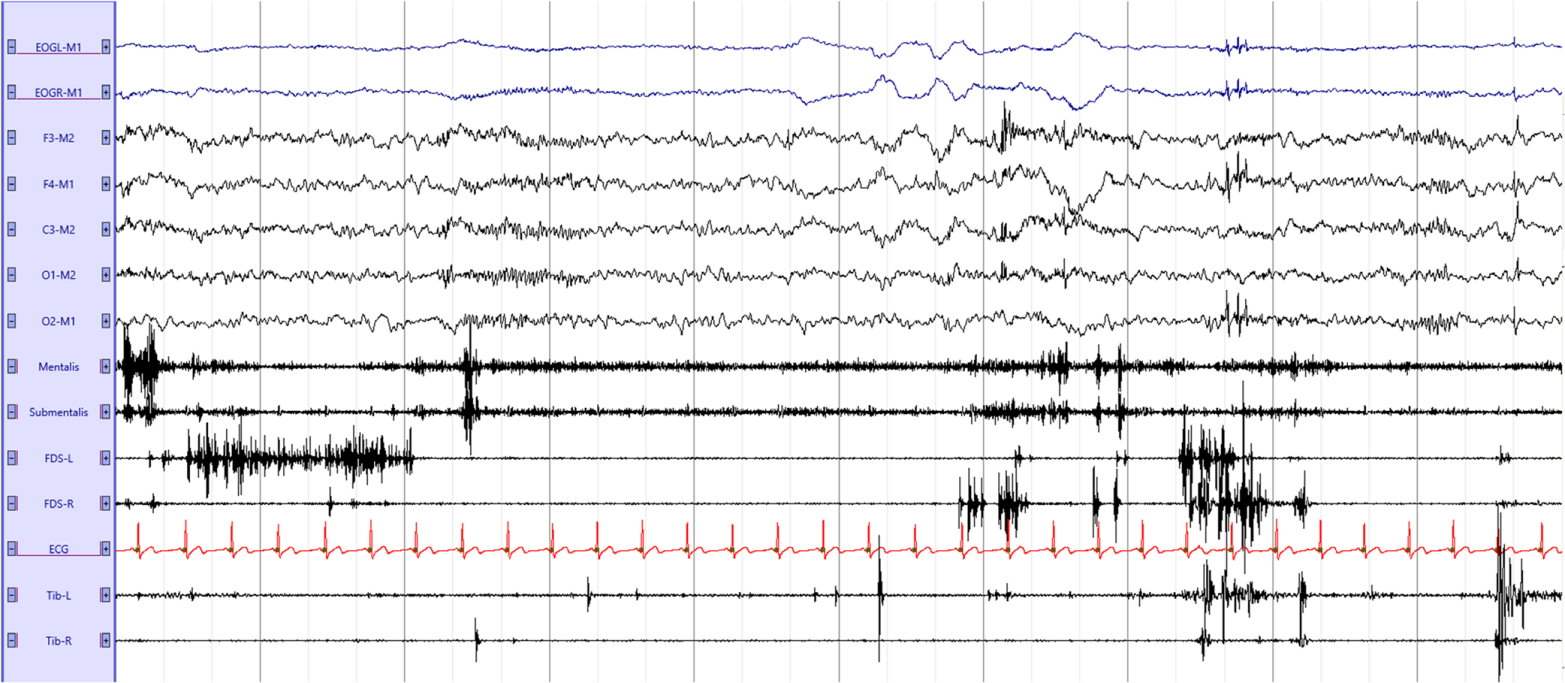
Typical example of 30 s of REM sleep in a patient with iRBD. It is possible to appreciate the presence of RWA in the EMG channels (mentalis, submentalis, bilateral FDS and bilateral tibialis anterior). EMG, electromyography; FDS, flexor digitorum superficialis; iRBD, isolated REM sleep behaviour disorder; REM, rapid eye movement; RWA, REM sleep without atonia.

The current gold standard for diagnosing RBD is video‐polysomnography (v‐PSG), which is necessary to confirm REM sleep without atonia (RWA) (American Academy of Sleep Medicine 2023). Figure 1 shows a typical example of RWA in a PSG. According to recent guidelines from the International RBD Study Group (IRBDGS), a definitive diagnosis of RBD also requires the documentation of at least one RBD episode—defined as one or more motor events and/or vocalisations associated with dream enactment—during v‐PSG (Cesari et al. 2022). These guidelines have received partial endorsement from the World Sleep Society (Schenck et al. 2023).

Currently, the detection of RWA and the identification of RBD episodes rely on visual and manual analyses (Cesari et al. 2022; Troester et al. 2023). These processes are not only time‐consuming but also susceptible to inter‐rater variability (Bliwise et al. 2018). As a result, incidental cases, that is, patients undergoing sleep studies for unrelated conditions, may go undetected. Additional factors such as the limited number and accessibility of specialised sleep labs and low awareness of the condition further contribute to the under‐diagnosis of RBD (Murray et al. 2024; White et al. 2012).

Given the potential availability of future neuroprotective or neuromodulatory treatments, effective screening tools for iRBD are key. Such tools could enable the identification of the disorder in the general population, addressing current resource limitations. This manuscript explores recent advancements in RBD screening methodologies, critically evaluates their limitations and highlights their potential for future implementation.

## 2 Questionnaires

As a screening tool, a number of RBD questionnaires, such as RBD Screening Questionnaire (RBDSQ) (Stiasny‐Kolster et al. 2007), RBD Single Question (RBD1Q) (Postuma et al. 2012), Innsbruck RBD Inventory (RBD‐I) (Frauscher, Ehrmann, et al. 2012), Mayo Sleep Questionnaire (MSQ) (Boeve et al. 2011) and RBD Questionnaire‐Hong Kong (RBDQ‐HK) (Li et al. 2010), have been developed to facilitate the identification of possible RBD cases when the gold standard polysomnographic assessment is not accessible or suitable, for example, in large scale community epidemiological surveys. However, the diagnostic value of RBD questionnaires has been mostly assessed and validated in clinical settings, with only a few exceptions of validation in community‐based samples (Boeve et al. 2013). These questionnaires have been translated into different languages (Marelli et al. 2016; You et al. 2017), validated across different sleep centres (Postuma et al. 2012) and widely used around the world. They could also be used to monitor disease progression and treatment responses (Li et al. 2010), but this aspect is outside the scope of this paper and will not be addressed.

In the clinic, RBD questionnaires have shown high sensitivity (> 80%) in predicting clinical v‐PSG confirmed RBD (Boeve et al. 2011; Frauscher, Ehrmann, et al. 2012; Li et al. 2010; Postuma et al. 2012; Stiasny‐Kolster et al. 2007), while the specificity varied greatly, ranging from 56% to 87%. A recent paper examined the performance of multiple questionnaires (RBDSQ, RBD1Q and RBD‐I) in a bi‐centric study on patients presenting to a sleep lab for the first time prior to any clinical interview or assessment and observed that their specificities were consistently low (48.1%–67.4%) (Stefani, Serradell, et al. 2023). In addition, even with the strict ‘all questionnaires positive’ criterion, the specificity was still only moderate, at around 77%. In other words, there is a sizable proportion of false positive cases as based on questionnaire screening, so called RBD mimics, such as nocturnal behaviours related to sleep apnea, nightmare, medications and NREM parasomnia. On the other hand, when RBD questionnaires were applied in epidemiological surveys, the calculated prevalence of possible RBD (pooled estimate equals 5.65%) (Cicero, Giuliano, Luna, et al. 2021; Mahlknecht et al. 2015; Nomura et al. 2015; Shprecher et al. 2019; Wong et al. 2016; Yao et al. 2019) was considerably higher than the prevalence of v‐PSG‐confirmed RBD (close to 1%) (Chiu et al. 2000; Cicero, Giuliano, Sgroi, et al. 2021; Cicero, Giuliano, Luna, et al. 2021; Haba‐Rubio et al. 2018; Kang et al. 2013; Lee et al. 2023; Pujol et al. 2017; Sasai‐Sakuma et al. 2020). The reason for this gap between possible and definite RBD, except for the suboptimal specificity that has been described, is related to the relatively low prevalence of RBD in the general population, which has greatly reduced the positive predictive values (PPV) of these questionnaires.

In conclusion, RBD questionnaires, while showing moderate to high sensitivity, moderate specificity but rather low PPV values especially in a community setting, thus indicating that there is a need to improve this tool for possible screening of patients with iRBD.

## 3 Can Automatic Algorithms Improve V‐PSG Detection of RBD?

Screening for RBD in the sleep lab is also of crucial importance, as patients with incidental RBD diagnosis might be missed during a routine PSG performed for other reasons, due to lack of time for manual RWA quantification and visual analysis of movements identified by video.

### 3.1 Automatic Methods to Score REM Sleep Without Atonia

RWA is defined as excessive EMG activity during REM sleep and is an essential feature for diagnosing RBD. Current RWA quantification methods are rule‐based and rely on visual inspection and manual scoring of EMG activity in the chin and limbs (Cesari et al. 2022; Troester et al. 2023). The most used approaches, including the Montréal, SINBAR and Mayo Clinic methods (Frauscher, Iranzo, et al. 2012; McCarter et al. 2014; Montplaisir et al. 2010), categorise muscle activity as tonic, phasic, or ‘any’ based on the amplitude or burst duration, and employ predefined thresholds for RBD identification. Both the ICSD‐3‐TR (American Academy of Sleep Medicine 2023) and IRBDSG guidelines (Cesari et al. 2022) recommend the SINBAR montage and cut‐offs for RBD diagnosis. An open‐source software for computing the SINBAR metrics is available, featuring high reliability with manual scoring, though it requires input data to be in a specific PSG‐software format (Röthenbacher et al. 2022). Several other automated approaches, extracting EMG metrics in fixed‐length epochs (Burns et al. 2007; Frandsen et al. 2015) or employing machine learning (ML) (Cesari et al. 2019; Kempfner et al. 2014; Rechichi et al. 2021), were developed to minimise variability in RWA metrics and offer promising solutions for standardised RWA quantification (Cesari and Rechichi 2024). Among these, the REM Atonia Index (RAI) (Ferri et al. 2010, 2008) is the most validated automatic RWA quantification method and therefore emerges as a reliable screening tool for RBD identification, though currently there is no available open‐access software for deriving it. In the future, openly sharing the code of automatic and validated RWA quantification methods will be essential to ensure the quality of the implementation and reproducibility across labs.

### 3.2 Advanced Electroencephalogram (EEG) Analyses to Identify Patients With iRBD

Advanced sleep EEG analyses have been shown capable of predicting mortality, dementia, and long‐term neurologic and cognitive outcomes (Djonlagic et al. 2021; Ganglberger et al. 2022; Paixao et al. 2020; Sun et al. 2024; Ye et al. 2020; Younes et al. 2021). These techniques also provide insights into RBD, uncovering cortical abnormalities that standard polysomnographic analysis cannot detect (Fantini et al. 2003; Iranzo et al. 2010). Beyond spectral power assessments, advanced EEG approaches—such as microstate segmentation, connectivity and coherence analyses, complexity measurements and dynamic time‐frequency evaluations—identify subtle cortical slowing, altered spindle dynamics, and abnormal oscillatory coupling in both REM and NREM sleep of RBD patients (Christensen, Kempfner, et al. 2014; O'Reilly et al. 2015).

Notably, RBD patients show, in sleep EEG, shifts toward slower frequencies and reduced beta/gamma power, potentially signalling early neurodegenerative processes and a heightened risk of progressing to PD or dementia (Fantini et al. 2003; Iranzo et al. 2010; Massicotte‐Marquez et al. 2005). Moreover, recent studies have highlighted that micro‐sleep instability, characterised by rapid fluctuations of sleep–wake micro‐architecture, may predict the emergence and progression of RBD (Cesari, Christensen, et al. 2021). Additionally, longitudinal analyses of EEG features over years can illuminate the subtle temporal progression of cortical changes as RBD advances toward neurodegeneration (Angerbauer et al. 2024; Schreiner et al. 2019).

Advanced EEG analyses, however, can be applied not only to study sleep EEG, but also resting state EEG, which could also be potentially seen as a screening tool on its own. Evidence shows that patients with iRBD are characterised by loss of delta‐band functional connectivity (Sunwoo et al. 2017), alterations in microstate features (Peng et al. 2021), alterations in spectro‐spatial patterns (Park et al. 2024) and slowing (Rodrigues Brazète et al. 2016, 2013; Ruffini et al. 2019).

In general, research works indicate the potential of advanced analyses applied to both sleep and wake EEG to identify patients with iRBD.

### 3.3 Machine and Deep Learning Algorithms Combining Different Electrophysiological Signals

In addition to RWA, an abundance of electrophysiological markers derived from PSG has been associated with RBD, including EEG, electrooculogram (EOG) (Christensen et al. 2021) and electrocardiogram (ECG) (Sorensen et al. 2012). This supports the application of ML and deep learning (DL) to PSG data, aiming at integrating multiple electrophysiological markers to develop a robust and precise automatic analysis system that could be employed as a decision support system or to evaluate severity and phenoconversion risk.

ML algorithms have been utilised not only to improve characterisation of RWA, as previously mentioned (Cesari et al. 2019; Kempfner et al. 2014; Rechichi et al. 2021), but also to summarise EEG and EOG abnormalities (Cesari, Christensen, et al. 2021; Christensen, Zoetmulder, et al. 2014; Hansen et al. 2013; Rechichi et al. 2022), and to analyse PSG data in a unified framework to detect RBD (Cooray et al. 2021, 2019; Salsone et al. 2022). Moreover, DL has also been used as a modelling strategy (Brink‐Kjaer et al. 2022; Feuerstein et al. 2024; Gunter et al. 2023), which avoids directly having to define and extract abnormal activity. One of the more recent DL approaches (Feuerstein et al. 2024) modelled RBD from an extracted hypnodensity—a probabilistic representation of sleep stages—based on a validated algorithm, which highlights the usefulness of reduced but information‐rich representations of PSG data. The use of foundational models for RBD detection is yet to be explored, but may improve detection performance by allowing complex data mapping while addressing RBD data limitations.

### 3.4 Automatic Video Analysis

RBD is characterised by dream enactment behaviours during REM sleep, and more generally by the loss of motor inhibition during this sleep stage. Movements in patients with RBD range from simple jerks to complex behaviours. While complex behaviours are infrequent, simple, brief movements or jerks occur during most REM cycles—every few seconds to minutes—and independent of respiratory‐related arousals (Bugalho et al. 2017; Frauscher et al. 2007; Manni et al. 2009; Mariño et al. 2025). This characteristic forms the foundation for video‐based algorithms to detect RBD.

The application of computer vision for RBD detection is a developing field, with three key studies conducted to date, all in sleep laboratory settings. Two studies by the Innsbruck group utilised a 3D time‐of‐flight video system (Cesari, Ruzicka, et al. 2023; Waser et al. 2020), while another study by the Stanford and Mount Sinai groups analysed retrospective 2D video data from clinical vPSG (Abdelfattah et al. 2025).

The first study by the Innsbruck group examined 40 iRBD patients and 64 controls with various sleep disorders, finding that brief leg movements (< 2 s) were approximately 5 times more frequent in iRBD (Waser et al. 2020). This feature alone differentiated iRBD from controls with 90.4% accuracy. Other features investigated were 3D extent (body area involved in the movement) and 3D intensity (speed). The second study (53 cases, 128 sleep clinic controls) used ML to analyse movements across all body regions (Cesari, Ruzicka, et al. 2023). In this dataset, a linear regression model using only 2 features—movement rate (frequency) and movement ratio (proportion of REM sleep spent in movements)—yielded an accuracy of 86.6%, with short movements providing the highest discriminative power.

The study by Stanford‐Mount Sinai of 81 iRBD cases and 91 controls adopted a similar ML model but differed in two ways: it was retrospective, using 2D clinical video, and incorporated the following features: movement magnitude (area), velocity (speed) and immobility ratio (Abdelfattah et al. 2025). Accuracies ranged from 84.9% (two features) to 87.2% (five features) when analysing movements of all durations. However, consistent with the Innsbruck studies, focusing solely on short movements achieved the highest accuracy (91.9%).

Although limited in number, existing studies consistently highlight the diagnostic potential of brief movements during REM sleep as a robust digital biomarker. However, current methodologies rely on labour‐intensive manual annotation of REM sleep periods. Future research should focus on developing fully automated algorithms that integrate REM sleep detection with movement analysis. Beyond screening, video‐based models offer an exciting opportunity to objectively monitor clinical severity over extended periods in the home environment. This capability could provide valuable insights into treatment efficacy and disease progression, addressing a critical gap in the long‐term management of RBD and the development of new symptomatic therapies.

## 4 Actigraphy

Actigraphs are devices typically worn on the wrist containing accelerometers, which detect and record acceleration. Through acceleration, the amount and pattern of movements can be calculated, from which periods of activity and rest can be inferred by means of *ad hoc* algorithms. Actigraphy is a relatively inexpensive, easy to implement diagnostic test which is suitable for rest/activity monitoring over prolonged periods of time, usually weeks, as a surrogate marker of the sleep/cycle.

Patients with RBD, either isolated forms or associated with PD, DLB or MSA, exhibit increased nocturnal muscular activation during REM sleep (Schenck and Mahowald 2002), and in iRBD this tends to increase over time (Iranzo et al. 2009). RBD movements are more brisk and violent compared to movements and behaviours observed upon ‘normal’ arousals (De Cock et al. 2007). This heightened activity provides a rationale for using accelerometers to differentiate between individuals with RBD and healthy controls. Moreover, as a neurodegenerative condition directly involving the circuits of sleep regulation, iRBD features a progressive destructuring of sleep itself. This alterations not only affect nocturnal sleep but are also evident at the level of the rest/activity rhythm (Feng et al. 2020; Filardi et al. 2020).

Actigraphic data can be employed as ‘digital biomarker’ for RBD (Gnarra, Wulf, et al. 2023; Stefani and Cesari 2023). Recent, preliminary studies have demonstrated that by extracting features from whole‐night accelerometric recordings with data‐driven approaches it is possible to predict the presence of RBD using ML methods, thus distinguishing patients with iRBD (Brink‐Kjaer, Winer, et al. 2023; Brink‐Kjaer, Gupta, et al. 2023) or RBD associated with PD (Raschellà et al. 2023), from controls. Although very promising, these approaches need further implementation, fine‐tuning and optimization, especially for applicability in the general population. As a further step, their accuracy in discriminating RBD from other sleep disorders (e.g., sleep‐related breathing disorders, nocturnal seizures or disorders of arousal) will need to be assessed.

Implementing actigraphy‐based quantitative methodologies and validating them in large population‐based, prospective studies will enable the use of actigraphy not only as a screening and diagnostic biomarker but also for a prognostic profiling of iRBD patients, to identify those at higher risk of phenoconversion to an overt synucleinopathy. Also, quantitative actigraphy‐derived metrics might be useful for patients' follow‐up and response to treatment evaluation, both in clinical practice and research, and also to test putative neuroprotective agents or interventions. Improving the reliability and applicability of actigraphy will facilitate its integration into personalised medicine, long‐term monitoring and clinical research on RBD.

## 5 Nearables: A Promising Tool for Screening RBD

The advent of nearable technologies has opened new avenues for screening sleep disorders, including RBD. Nearables, defined as unobtrusive devices capable of monitoring physiological and environmental parameters (Rienzo and Mukkamala 2021), offer a practical alternative to traditional PSG. Devices such as bed sensors, smart mattresses and contactless radar‐based systems have demonstrated the ability to detect abnormal motor activity and physiological markers indicative of RBD (Gnarra, Wulf, et al. 2023). Although none of these technologies has yet been validated for identifying RBD, many advances have been made recently. Radar technologies combined with ML algorithms have proven effective at predicting PD from nocturnal breathing signals (Yang et al. 2022). Technologies such as sensorised mattresses have been used for the automatic classification of body positions (Matar et al. 2020) and complex motor behaviours (Deng et al. 2024) during sleep that characterise REM sleep disturbances.

Nearables provide several advantages over standard PSG. Their hands‐off approach eliminates the need for direct interaction with the user, making them particularly suitable for elderly populations or individuals with limited cognitive or physical abilities. By enabling continuous, long‐term monitoring in home environments, nearables facilitate sleep data collection under realistic conditions without requiring hospital setups (Breuss et al. 2024). Additionally, nearables can generate multi‐modal data streams, including motion, cardiac and respiratory signals. For instance, smart bed systems with piezoelectric sensors can detect micro‐movements, heart rate and respiratory patterns, while radar‐based systems can monitor gross motor activity (Ravindran et al. 2023) and perform reliable automated sleep staging (He et al. 2025) without physical contact. These features make nearables a user‐friendly and scalable solution for large‐scale screening.

However, nearables have notable limitations compared to PSG. While they simplify data collection and reduce patient discomfort, they lack the precision of EEG and EMG‐based methods, which are critical for accurately detecting RWA/RBD and differentiating them from other sleep movement disorders. Additionally, nearables currently cannot capture brain activity or subtle physiological changes with the resolution required for diagnostic accuracy. The reliance on indirect measures introduces potential variability in data interpretation, which could lead to false positives or negatives. Furthermore, challenges such as limited standardisation, potential signal interference in shared sleeping environments, and the need for robust algorithms to minimise false positives remain significant (Gnarra, Breuss, et al. 2023).

At their current stage of development, nearables are most effective for screening purposes rather than for providing a definitive diagnosis of RBD. They represent a valuable addition to the field of sleep medicine, aligning with the ongoing shift toward home‐based monitoring. Their ability to combine convenience, scalability and multi‐modal data collection positions them as a future tool for early detection and tracking of RBD. While they cannot replace PSG in diagnostic settings, nearables hold significant potential to improve access to care and facilitate timely interventions for affected individuals.

## 6 Biological Markers

Currently, no fluid‐ or tissue‐based biological marker exists for screening RBD. However, iRBD itself serves as a highly specific clinical marker for early synucleinopathies (Joza et al. 2023; Postuma et al. 2019). In this context, iRBD can facilitate identifying biological markers of early synucleinopathies.

Pathological alpha‐synuclein species could be identified in individuals with iRBD in multiple peripheral tissues and fluids, including salivary glands (Vilas et al. 2016), olfactory mucosa (Kuzkina, Rößle, et al. 2023; Zheng et al. 2024), gastrointestinal mucosa (Sprenger et al. 2015), stool (Schaffrath et al. 2023), skin (Doppler et al. 2022; Kuzkina, Panzer, et al. 2023; Kuzkina, Rößle, et al. 2023) and blood (Arnaldo et al. 2023; Kluge et al. 2024; Okuzumi et al. 2023; Ying et al. 2024; Zheng et al. 2023), as well as biospecimens from the central nervous compartment (Poggiolini et al. 2022; Siderowf et al. 2023), highlighting the strong link to incipient alpha‐synucleinopathies. The detectability of additional protein aggregates, such as amyloid or tau, in iRBD may indicate a faster phenoconversion and more pronounced cognitive symptoms (Fernandes et al. 2024). Similarly, elevated levels of neurofilament light chain (Nfl) indicate a more likely phenoconversion to MSA (Park et al. 2023). Fewer individuals with RBD unmasked by antidepressant treatment exhibited pathological alpha‐synuclein species in skin biopsies compared to iRBD not related to antidepressants, enabling insights into underlying causes of RBD (Biscarini et al. 2024).

However, changes extend beyond the mere detectability of altered alpha‐synuclein or additional neuronal proteins, as individuals with iRBD already exhibit signs of exacerbated inflammation (Farmen et al. 2021; Hällqvist et al. 2024; Laguna et al. 2021) and gut microbiome dysbiosis (Huang et al. 2023), accompanying early neurodegenerative processes (Glass et al. 2010). Those changes are also reflected in an altered composition of microRNAs, which can be detected in peripheral blood (Soto et al. 2022; Yu et al. 2024).

Current research focuses on using iRBD as a specific clinical entity to identify biological markers of early (alpha‐synuclein associated) neurodegeneration rather than on screening for biological markers of iRBD per se. This might change in the future, as improved understanding of iRBD pathogenesis in the early stages might lead to the identification of biological markers of iRBD useful as screening tools.

## 7 Other Screening Markers

In addition to the possible screening markers reported in the previous sections, biomarkers of neurodegeneration have been investigated in the iRBD population and are worth mentioning. Alterations in voice have been reported in patients with iRBD and might be used as screening tools, both with standardised protocols (Arora et al. 2021; Jeancolas et al. 2022; Rusz et al. 2021, 2016), as well as with free speech recorded during calls (Illner et al. 2024). Smartphone motor testing, performed with standardised exercises, has also been shown to be useful to distinguish patients with iRBD from controls (Arora et al. 2018). Furthermore, gait changes detected with wearable sensors or automatic video analyses have been investigated and showed promising discrimination abilities (Del Din et al. 2019; Ma et al. 2021; Sarasso et al. 2024). Similarly, analysis of timed‐up‐and‐go tests recorded with wearable technologies allowed for the discrimination of patients with iRBD from controls (Zatti et al. 2024). Moreover, two studies investigated EEG headbands to identify biomarkers of iRBD (Levendowski et al. 2022; Possti et al. 2024). Of note, these biomarkers aim at detecting subtle signs of neurodegeneration, which are present in iRBD (being this condition a prodromal synucleinopathy), but do not aim at individuating iRBD per se. Thus, a future employment of these instruments as screening methods would be useful in detecting early synuclein‐related neurodegeneration, but would probably not be specific for iRBD and would not detect RBD associated with diseases different from alpha‐synucleinopathies.

## 8 Discussion

**FIGURE 2:**
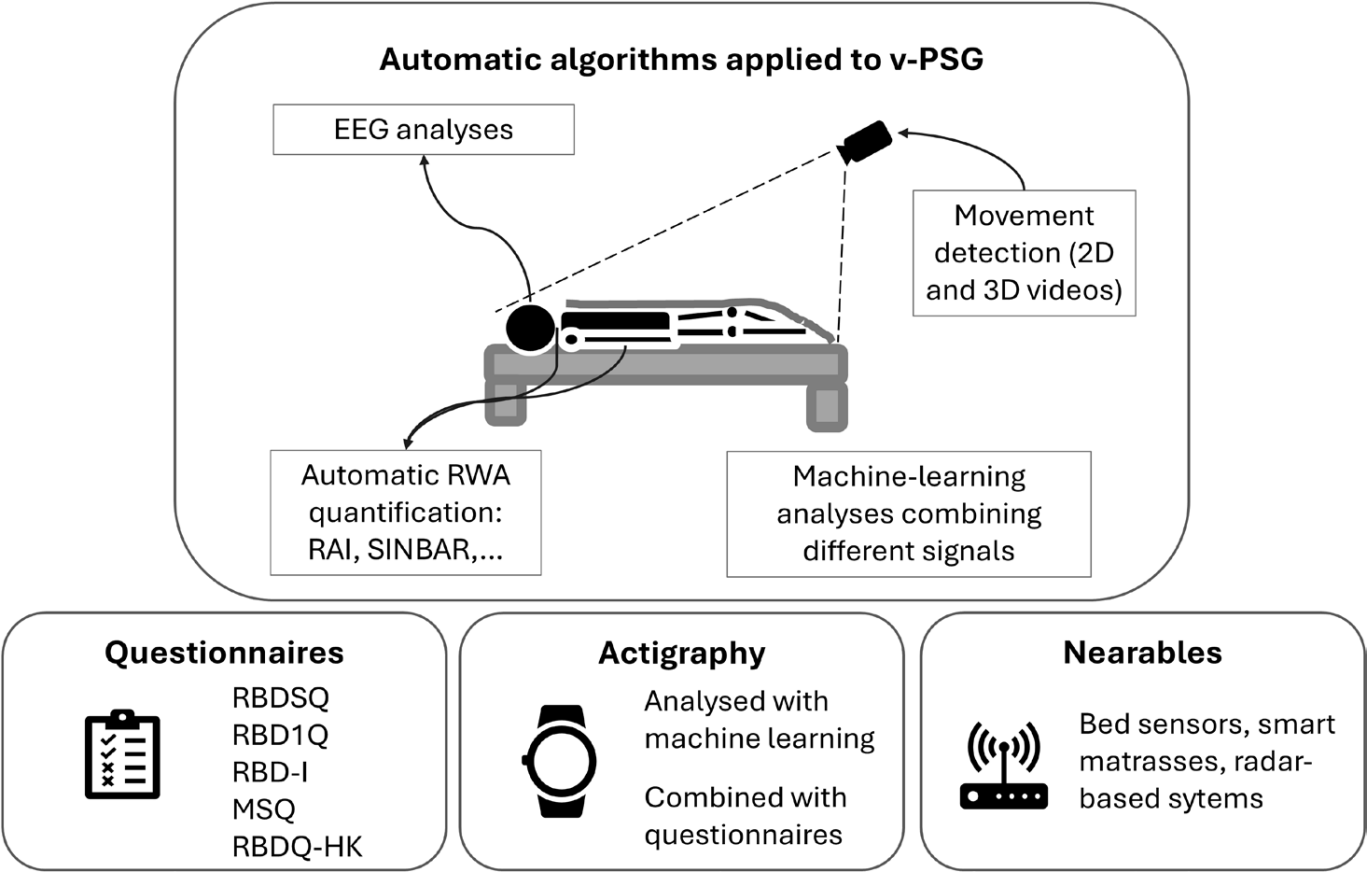
Overview of the screening methods for patients with iRBD described in this manuscript. Please note that the figure does not report biological markers (Section 6) and other markers (Section 7), because those markers might not be specific for iRBD, as they aim at detecting neurodegeneration independently from the presence of RBD.

In view of upcoming neuroprotective or neuromodulatory treatments, effective screening tools for iRBD are of fundamental importance. We reviewed the most important recent advancements in this research area, a timely topic of growing importance. Figure 2 shows a graphical overview of the screening tools specific for iRBD described in this manuscript.

Besides the single screening methodologies presented previously, it is worth considering the potential of their combination and multistep screening approaches. Questionnaires are the easiest and most convenient tool to be used as a first step in a multistep screening approach, due to their favourable sensitivity and NPV, as proposed previously (Postuma et al. 2016). The combination of questionnaires with actigraphs has already shown promising results, as these modalities led to an increase of the specificity to 100% (Brink‐Kjaer, Gupta, et al. 2023). Furthermore, previous multistep‐approach studies have been shown to be useful for a precise identification of patients with iRBD (Pujol et al. 2017; Seger et al. 2023; Wang et al. 2022). In brief, inexpensive and non‐invasive tests with high sensitivity could be used as a first step, for example, questionnaires (±actigraphy), followed by relatively expensive and labour‐intensive tests with high specificity, such as gold‐standard v‐PSG, or biological markers to stratify RBD patients.

Concerning v‐PSG analyses, automatic and validated RWA quantification algorithms should be implemented as soon as possible in clinical PSG‐software solutions. So far, only one PSG‐software includes a validated RWA quantification algorithm (Frauscher et al. 2014), but it still requires manual artefact removal (Cesari, Heidbreder, et al. 2021). However, due to the low amount of artefacts affecting these muscles, automatic analysis of FDS alone can be used as a screening tool (Cesari, Heidbreder, et al. 2023), pending further confirmation in larger cohorts.

ML approaches combining different electrophysiological biomarkers have shown promising results but lack sufficient validation, which would likely require strong collaboration efforts to test generalisability on large multi‐centre datasets, comparison to simpler automatic RWA methods, and study of night‐to‐night variability of derived measures.

Integration of automatic video analyses and electrophysiological sleep biomarkers has not been investigated so far, and deserves attention in the near future. While automatic analyses of in‐lab v‐PSG could be useful in order not to miss incident subclinical RBD patients and as a second screening step following questionnaires, current technological developments allow good quality sleep recordings in home environments (Green et al. 2022). So far, however, there is only limited research on the identification of patients with iRBD from home sleep recordings.

Future research for the development of more accurate screening tools should also consider sex and ethnicity related aspects. For example, previous studies suggest that there are sex differences in muscular activity, motor events (Bugalho and Salavisa 2019), demographics and dream‐related behaviours (Li et al. 2023). These should be considered when developing new screening tools, to have high sensitivity and specificity in both sexes. Nothing is known about possible ethnic differences in the clinical presentation of iRBD. Future studies should investigate ethnic aspects and take them into consideration when developing novel screening methods.

In conclusion, future screening methods for RBD will likely use multistep approaches combining subjective and objective data, the latter integrating information from wearables and nearables used in the home environment. Biological markers will probably improve risk stratification. ML and DL methods to use this information thoroughly are expected to drastically improve screening of RBD in the general population in the near future.

## Author Contributions

**Matteo Cesari:** writing – original draft, writing – review and editing, conceptualization. **Andreas Brink‐Kjaer:** writing – original draft, writing – review and editing. **Emmanuel During:** writing – original draft, writing – review and editing. **Wolfgang Ganglberger:** writing – original draft, writing – review and editing. **Oriella Gnarra:** writing – original draft, writing – review and editing. **Bei Huang:** writing – original draft, writing – review and editing. **Michael Sommerauer:** writing – original draft, writing – review and editing. **Pietro Luca Ratti:** writing – original draft, writing – review and editing. **Irene Rechichi:** writing – original draft, writing – review and editing. **Yun Kwok Wing:** writing – original draft, writing – review and editing. **Ambra Stefani:** conceptualization, writing – original draft, writing – review and editing, supervision.

## Conflicts of Interest

Matteo Cesari received consultancy fees from Xtrodes Ltd. Michael Sommerauer received funding from the program ‘Netzwerke 2021’, an initiative of the Ministry of Culture and Science of the State of Northrhine Westphalia, and from the Federal Ministry of Education and Research (BMBF) within the framework of the funding programme ACCENT (funding code 01EO2107). Yun Kwok Wing received personal fees from Eisai Co. Ltd., for delivering a lecture, and sponsorship from Lundbeck H.K. Ltd. and Aculys Pharma Inc., which are not related to the current manuscript. The other authors declare no conflicts of interest relevant for this manuscript.

## Data Availability

Data sharing not applicable to this article as no datasets were generated or analysed during the current study.
